# A Mixed Afternoon in October

**Published:** 1908-12-05

**Authors:** 


### The Practitioner's Relaxations.

### A MIXED AFTERNOON IN OCTOBER.

Two p.m. Five patients (of the small farming class) to see. Yes, I might do them and have an hour's shooting as well. Soon I had my gun strapped to the frame of a bicycle, the game bag on the carrier beside my professional bag, and, with a couple of dozen cartridges in a coat pocket, was off, a red setter racing alongside.

Arrived near the home of patient No. 1, I got off, and placing the cycle beside a fence called the dog (trained by long experience). He took his seat and did sentry duty beside the bicycle until I returned. I saw patient No. 1 and inquired of a male relative if there were any partridge about this year. He said he had seen a covey early this morning about a mile further on. It was not in the direction of patient No. 2, but I decided to have a try. I cycled down the road for about a mile, and, coming to the place, unstrapped the gun, put the game bag over my shoulder, and, lifting the cycle over the road fence, directed the dog to try a field of roots.

Up he goes to windward and beats all along the headland and now dashes in. To right and left he goes, and then stands and looks in my direction. I hate that. When he looks to me for that wave of my hand to convey to him where to go next it means he has made nothing out of his nose. I signal him up to the left, and now a rabbit bolts down the headland. Bang! I've missed him clean. But there the dog is standing. As I approach, a cock pheasant gets up, clucking on the rise, and comes down immediately I fire. We leave the field and try the next, and next, and now we get on the track of the birds. The dog trails them two fields down, along a sheep path, and then loses scent. I look around. Some fields further away is a crop of potatoes, the leaf yet on the stalks. The birds may have flown there. They like burrowing in clay in most weathers, but to-day the sun is hot, and they are probably in the field of roots lower down. We enter this and draw it blank, and next go into the potato field. Lower down the dog stands. Up gets a snipe and I drop him. The noise of the shot arouses the attention of harvesters in a field a short distance away, and one, leaving his work, comes to watch me. I know he is the owner of the land I am on, and a client of mine too, so I continue to beat the potato field. The dog springs another snipe and I miss him. I now go towards the watcher, and see my dog, tail and head in one muscularly strained intent tonic spasm, glaring with excited eyes forward to a little rise in the open grass field. I raise a cautioning hand to the farmer and walk up. Whirr! Up they get. I mark the first bird and drop him and swing round to get at the rest. A bird passes before the sight. I wait. Two pass. I wait, and now two close together. I fire and get two. "Good shot, Doc!" Yes, I feel proud. It does not suit a doctor to show himself a duffer at anything before the eyes of those who are his potential patients or their friends. I would thus advise my readers never in the common field of pastimes, sport particularly, to attempt, before the eyes of those to whom they may one day have to give their best professional skill, to take a hand in any game unless they are certain of a creditable performance. There are those who will make their failure a weapon of speculative criticism on their professional skill.

I bagged the three birds, and finding the rest of the covey had flown into preserved ground, I bade the farmer good-day and returned to my bicycle. I re-strapped the gun and turned to visit patient No. 2. It was now 3.30. I rode hard, and reaching the home of patient No. 2, unslung my game bag and became the practitioner again. By four o'clock I was again on the road, and by 4.20 I had seen patient No. 3. I now was informed of another covey, and decided to put off my visit to the other two patients until it got duskish and too dark to shoot. Luck was in my way. I was freewheeling down an incline when in a wheat stubble field beside the road I saw what I thought was the raised head of a pheasant, or was it a withered thistle? I put the brake on and stopped and looked. "If it moves, I know it's not a thistle." Yes, I noticed a perceptible movement. I unslung the gun, laid the machine by the roadside, and crossed the fence. The dog immediately stood them; up they rose, a strong covey. I got two, right and left. The rest crossed the road. There was a potato field and a big patch of roots a hundred yards away, and I fancied the birds took cover there. I went down and got them up one by one until I had added five to the bag. It was now five o'clock, so I decided it was time to go and see my patients. By 6.10 I had visited them all, and by 7 o'clock I was back at home, with time for a pipe after my tea. There was little listlessness about me as I went to make up medicine for the patients I had seen, and I radiated my good spirits to those who arrived for evening surgery. I did not lose any fees by my fun, and had five brace of partridge, a cock pheasant, and snipe, and the knowledge that my nerve was never better.

